# Asthma increased in young adults from 2008–2016 despite stable allergic rhinitis and reduced smoking

**DOI:** 10.1371/journal.pone.0253322

**Published:** 2021-06-24

**Authors:** Styliana Vasileiadou, Linda Ekerljung, Anders Bjerg, Emma Goksör

**Affiliations:** 1 Department of Paediatrics, University of Gothenburg, Sahlgrenska Academy, Gothenburg, Sweden; 2 Krefting Research Centre, Institute of Medicine, University of Gothenburg, Gothenburg, Sweden; 3 Department of Women’s and Children’s Health, Karolinska Institutet, Stockholm, Sweden; 4 Martina Children’s Hospital, Stockholm, Sweden; Srebrnjak Children’s Hospital, CROATIA

## Abstract

**Background:**

Studies have produced inconsistent results on prevalence trends in asthma and allergic rhinitis (AR). We surveyed young adults about asthma in 2008 and 2016 and examined the impact of gender, AR and smoking.

**Methods:**

Thirty-thousand randomly selected subjects aged 16–75 years in Western Sweden received postal questionnaires in 2008 and 50,000 in 2016. This study is based on responders aged 16–25 years, 2,143 in 2008 and 2,484 in 2016.

**Results:**

From 2008–2016 current asthma increased from 9.3% to 11.5% (p = 0.014) and was significant in males without AR (aOR 1.83, 95% CI 1.09–3.07) and male smokers (aOR 3.02, 95% CI 1.12–8.13). In both years the risk of current asthma was reduced by growing up on a farm (aOR 0.26, 95% CI 0.81–0.84 and aOR 0.47, 95% CI 0.23–0.996), independent of a family history of asthma or allergy. AR did not differ significantly from 2008–2016 (22.5% vs 24.4%, p = 0.144). Current smoking decreased from 20.3% to 15.2% (p<0.001), especially in females (23.5% to 16.2%, p<0.001). Female smokers started smoking later and smoked fewer cigarettes in 2016 than 2008. In 2016, 4.8% of the cohort reported using electronic cigarettes. Of those, 60.7% also smoked tobacco and more than two-thirds who used both (67.2%) were heavy smokers.

**Conclusion:**

Current asthma increased in respondents aged 16–25 from 2008–2016, mainly among males without AR and male smokers. Current AR levelled off in this young population, while current smoking decreased among females.

## Introduction

During the second half of the 20th century, the prevalence of asthma and allergic rhinitis (AR) increased in most parts of the world, including Sweden [1, 2]. However, later studies have presented inconsistent results on prevalence trends. Some studies reported that the previous upward trends in asthma had reached a plateau, especially among children and adolescents [3, 4], but others showed opposite trends [5–7]. A recent 2008–2016 population-based study of people aged 16–75 in Western Sweden suggested that the increase was more prominent in young adults [8].

AR is one of the most common chronic diseases among young adults [1] and has been strongly associated with the development of asthma, especially in adolescence [3, 9], which suggests that any changes in the prevalence of AR could explain a parallel change in asthma. AR has continued to increase during the last few decades [4, 10], but it was recently reported that it had levelled off among young adults [11]. Studies have also reported that smoking increases the risk of both childhood asthma [12] and adult-onset asthma [9], so it could influence changes in the prevalence of asthma. In 2008 a surprisingly high prevalence of smoking was reported among young females in Western Sweden, which was compatible with the overall higher prevalence of asthma symptoms [3].

The aim of this study was to describe trends in asthma prevalence among young adults in Western Sweden from 2008–2016, with a special focus on the impact of gender, AR and smoking habits during the study period.

## Material and methods

### Study area and population

The West Sweden Asthma Study (WSAS) was a population-based, cross-sectional survey performed in the West Gothia region of southwest Sweden, including Gothenburg, which is Sweden’s second largest city. The survey comprised of two cohorts, WSAS I in 2008 and WSAS II in 2016, where a postal questionnaire was sent to randomly selected inhabitants aged 16–75, to 30,000 in WSAS I and to 50,000 in WSAS II. The study population demographics and response rates have been described in detail elsewhere [8]. This study is based on responders to postal questionnaires aged 16–25 in both cohorts. Clinical data and register data were not included in the present study.

### Questionnaires, definitions and covariates

Identical three-part questionnaires were used in both cohorts, consisting of a modified version of the Swedish OLIN study questionnaire, a Swedish version of the GA^2^LEN questionnaire as well as additional questions about smoking habits, occupational and environmental exposures, as previously described [13, 14]. Relevant questions for this present study can be seen in S1 Table.

The definitions of outcomes were as follows; Current asthma: Having physician-diagnosed asthma OR ever asthma AND at least one of the following variables: asthma medication or attacks of shortness of breath or recurrent wheeze or any wheeze. (‘‘Have you ever been diagnosed as having asthma by a doctor?”, ‘‘Have you now or have you ever had asthma?”, ‘‘Do you use asthma medication on a regular basis or as needed?”, “Have you had asthma symptoms within the last 12 months?”, “Do you usually have wheezing or whistling in your chest when breathing?”, “Have you had whistling or wheeze in the chest at any occasion in the last 12 months?”). Current AR: ‘‘Have you had problems with AR during the last 12 months?”. Ever AR: ‘‘Have you now or have you ever had allergic eye or nose problems (hay fever)?”

Questions on family history of asthma or allergy, whether their family had a farm for the first 5 years of their lives, current (in the last 12 months) or ever smoking, as well as on heavy smoking (5 cigarettes or more a day) were included, as were questions on current use of snus, a moist tobacco product that sits in the mouth, and ever use of snus during at least six months. The 2016 questionnaire also included use of e-cigarettes. Analyses were made for no, current or ever use of nicotine (tobacco smoking, snus or e-cigarettes). The questionnaires included questions on employment (student, employed or unemployed) and the highest level of education (primary and secondary school, high school or university). Moreover, urban living (living in Gothenburg or outside) and occupational exposure to gas, dust or fumes were included.

### Statistical analyses

The statistical analyses were conducted with SPSS, version 25.0 (IBM Corp, Armonk, NY, USA). Frequencies, cross-tabulations, chi-square and one-way analysis of variance were performed. Binary logistic regression was used to study the associations between the risk factors and the outcomes. The data are presented as percentages and adjusted odd ratios (aOR) with 95% confidence intervals (CI). A p-value of < 0.05 was considered statistically significant. Univariate analyses were made for potential risk factors and confounders (S2 Table). Two models of multiple logistic regressions were used. First, a basic demographic analysis, adjusting for the cohort, gender, age and where they lived. Second, a fully adjusted multivariate model, adjusted for the cohort, gender, age, where they lived, family history of asthma or allergy, growing up on a farm, current smoking, occupational exposure to gas, dust or fumes and education. When adjusting for age, two age groups were used (16–20 and 21–25 years). The interaction analyses used the basic demographic model and so did the analyses of the difference in smoking from 2008–2016, which were also adjusted for the educational level and unemployment.

### Ethical approval

Both WSAS I and WSAS II were approved by the Ethics Committee of the University of Gothenburg. Consent was obtained from all participants themselves, including 16- and 17-year olds, in concordance with the ethical approval. Thus, consent was not obtained from parents or guardians of 16- and 17-year olds in the study.

## Results

### Participation

The 2008 and 2016 cohorts generated 2,143 (50%) and 2,484 (35%) responses from residents aged 16–25 years, respectively, mean ages of 20.99 (range 17–25) and 21.12 (range 16–25) years (p = 0.092). The study population is described in Table 1.

**Table 1:** Characteristics of the study population in 2008 and 2016.

	2008		2016		
Variable	%	n = 2143	%	n = 2484	p-value
Age 21–25 years	55.0	1177/2141	56.9	1414/2484	0.191
Female gender	56.6	1213/2143	56.9	1413/2484	0.858
Urban living (Gothenburg)	57.7	1236/2143	48.7	1207/2478	**<0.001**
Growing up on a farm	6.7	142/2107	6.7	164/2438	1.000
Family history of asthma or allergy	49.3	1015/2059	52.7	1232/2337	**0.025**
Occupational exposure to gas, dust or fumes	17.1	367/2143	13.5	336/2484	0.122
Unemployed	4.7	100/2116	4.3	105/2469	0.474
Level of education:					0.067
Primary / Secondary school	19.7	411/2088	20.6	505/2457	
High school	54.7	1142	56.6	1390	
University	25.6	535	22.9	562	

The p-value indicates the statistical difference in the prevalence between the two study populations, and it is marked with bold where it is significant.

### Asthma and respiratory symptoms

Current asthma increased significantly from 2008–2016 (9.3% to 11.5%, p = 0.014). This was confirmed by the basic demographic model (aOR 1.27, 95% CI 1.04–1.53), but not by the fully adjusted model (aOR 1.20, 95% CI 0.98–1.48). The increase was not significant in males (7.6% to 10.1%, p = 0.060) or females (10.6% to 12.6%, p = 0.113).

**Fig 1:**
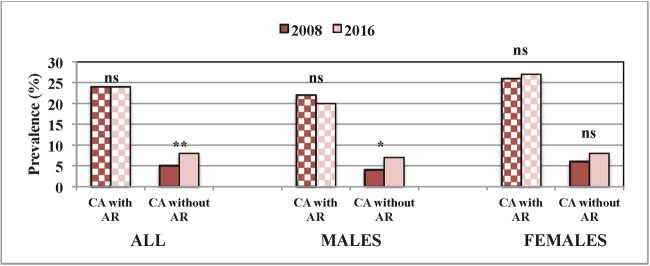
Current asthma in relation to allergic rhinitis and gender. The prevalence of current asthma (CA) among subjects with and without allergic rhinitis (AR) in 2008 and 2016, stratified for gender. *p<0.05, **p<0.01, ***p<0.001, ns = not significant.

There was a significant increase in current asthma without AR, from 4.9% to 7.5% (p = 0.002), in the fully adjusted model (aOR 1.43, 95% CI 1.05–1.96), especially in males (aOR 1.83, 95% CI 1.09–3.07) (Fig 1). Current asthma with AR remained stable (24.3% to 24.1%, p = 1.000).

Current asthma increased in current smokers (9.2% to 16.3%, p = 0.003), verified in the fully adjusted model (aOR 1.86, 95% CI 1.15–2.99) and only significantly in male smokers (aOR 3.02, 95% CI 1.12–8.13). Current asthma did not increase in non-smokers (9.3% to 10.8%, p = 0.142).

Ever asthma increased from 11.2% to 15.3% (p<0.001), in males (10.2% to 15.1%, p = 0.001) and females (12% to 15.5%, p = 0.009). It was confirmed by the basic demographic (aOR 1.43, 95% CI 1.20–1.70) and fully adjusted models (aOR 1.39, 95% CI 1.15–1.68). Ever asthma increased significantly among those without (6.5% to 10.9%, p<0.001), but not with (27.4% to 29.3%, p = 0.542), AR and was significant for both current smokers (9.7% to 20.6%, p<0.001) and non-smokers (11.4% to 14.5%, p = 0.005).

**Fig 2:**
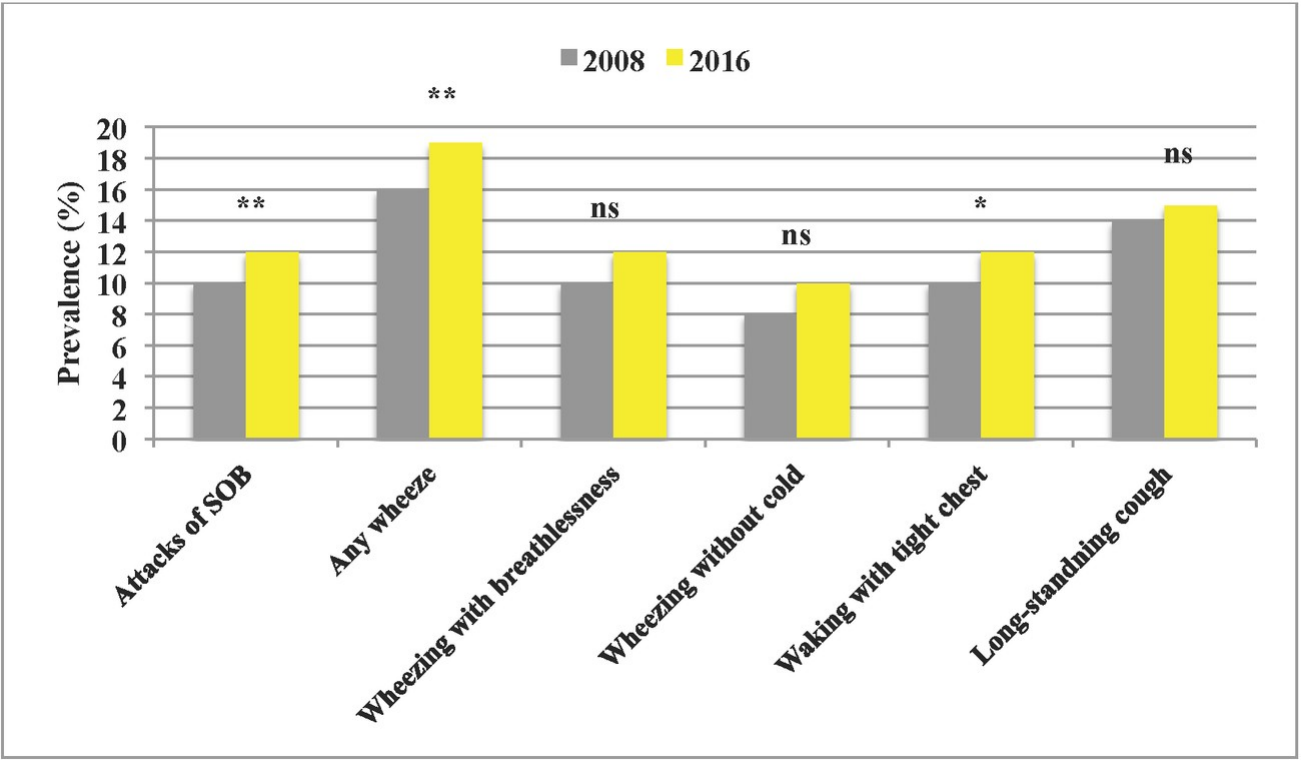
Respiratory symptoms in 2008 and 2016. The prevalence of respiratory symptoms during the last 12 months in 2008 and 2016. SOB = shortness of breath, *p<0.05, **p<0.01, ***p<0.001 and ns = not significant.

Respiratory symptoms seemed to increase (Fig 2), significantly among males but not females (S3 Table). Asthma medication during the last 12 months did not differ (9.6% to 10.8%, p = 0.190) irrespectively of gender.

### Risk factors for current asthma

**Fig 3:**
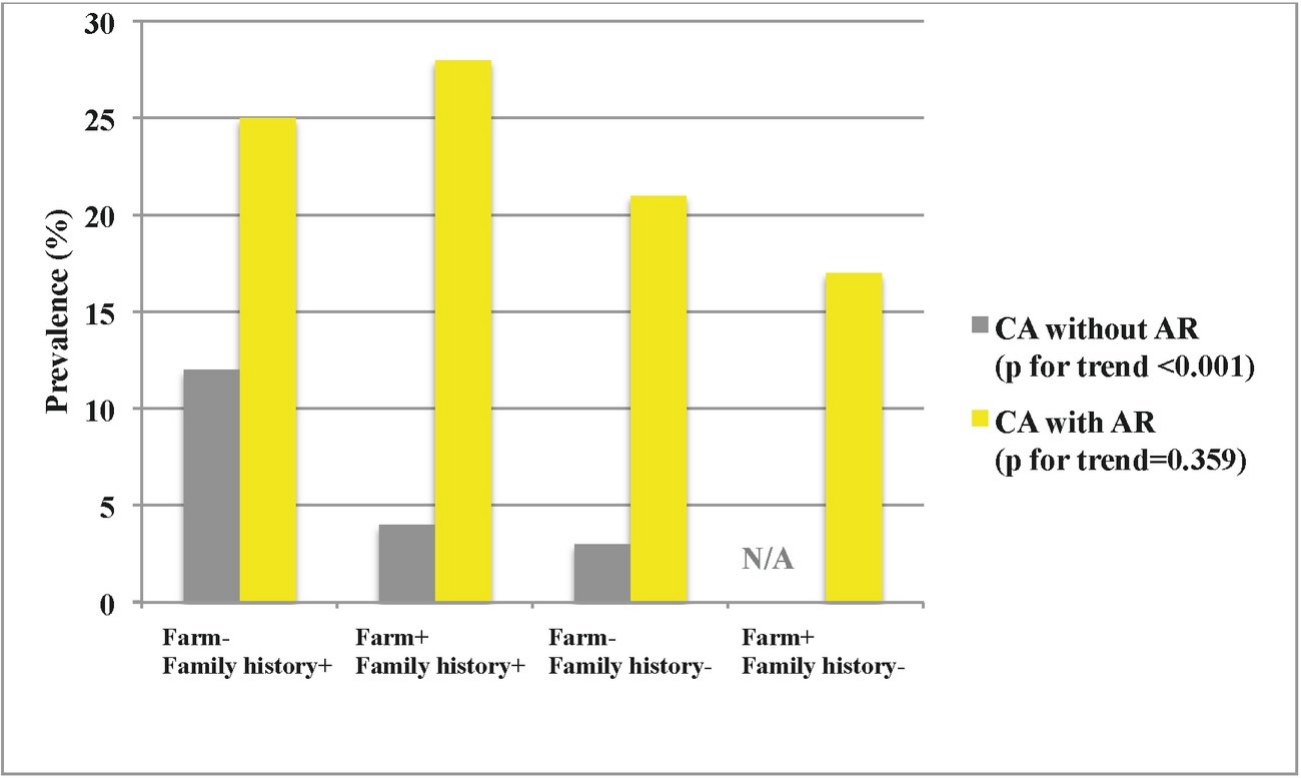
Current asthma and farm living. The prevalence of current asthma (CA) in 2016 among subjects with and without allergic rhinitis (AR), in relation to growing up on a farm and a family history of asthma or allergy. N/A = not applicable.

The independent risk factors for current asthma are shown in Table 2 and that growing up on a farm reduced the risk in both study years. In the fully adjusted model for 2016, growing up on a farm reduced the risk of current asthma for those without AR (aOR 0.21, 95% CI 0.05–0.88). This is illustrated in Fig 3 in relation to family history. Farm living did not lower the risk for those with AR.

**Table 2:** Independent risk factors for current asthma in 2008 and 2016.

	2008 n = 199	2016 n = 286
Variable	aOR (95% CI)	aOR (95% CI)
Female gender	**1.42 (1.02–1.98)**	1.18 (0.89–1.56)
Age 21–25 years	0.84 (0.58–1.22)	1.23 (0.89–1.69)
Family history of asthma or allergy	**3.14 (2.21–4.47)**	**4.03 (2.90–5.60)**
Current smoking	0.95 (0.64–1.42)	1.39 (0.98–1.99)
Growing up on a farm	**0.26 (0.81–0.84)**	**0.47 (0.23–0.996)**
Rural living (outside Gothenburg)	0.87 (0.62–1.21)	1.09 (0.82–1.45)
Occupational exposure to gas, dust or fumes	1.12 (0.74–1.71)	**1.47 (1.03–2.10)**
Level of education:		
Primary / Secondary school	1.0	1.0
High school	1.03 (0.67–1.58)	1.12 (0.77–1.63)
University	1.16 (0.68–2.00)	0.73 (0.44–1.21)

Bold indicates statistical significance. aOR = adjusted odds ratio.

We found significant interactions between family history and current AR in 2016 (p<0.001), but not 2008 (p = 0.112), and current smoking and gender in 2008 (p = 0.048), but not 2016 (p = 0.585).

### Allergic rhinitis

No significant change in current AR was seen (22.5% to 24.4%, p = 0.144), confirmed by the basic demographic model (aOR 1.11, 95% CI 0.97–1.27) and fully adjusted model (aOR 1.03, 95% CI 0.88–1.20), both in males (20.5% to 23.7%, p = 0.095) and females (24% to 24.8%, p = 0.617). Ever AR increased from 28% to 32% (p = 0.003), confirmed by the basic demographic model (aOR 1.20, 95% CI 1.06–1.37), but not the fully adjusted model (aOR 1.14, 95% CI 0.99–1.33). That change was significant among males (26.9% to 33%, p = 0.003), but not females (28.8% to 31.2%, p = 0.186).

### Risk factors for AR

The independent risk factors for current AR are shown in Table 3. Growing up on a farm reduced the risk of ever AR, both in 2008 and 2016 (aOR 0.55, 95% CI 0.32–0.92 and aOR 0.61, 95% CI 0.39–0.96, respectively).

**Table 3:** Independent risk factors for current allergic rhinitis in 2008 and 2016.

	2008 n = 482	2016 n = 605
Variable	aOR (95% CI)	aOR (95% CI)
Female gender	0.995 (0.79–1.26)	0.98 (0.79–1.21)
Age 21–25 years	1.14 (0.86–1.49)	**0.75 (0.59–0.97)**
Family history of asthma or allergy	**3.93 (3.07–5.04)**	**4.59 (3.62–5.83)**
Current smoking	0.95 (0.71–1.27)	1.17 (0.88–1.57)
Growing up on a farm	0.67 (0.39–1.13)	0.67 (0.41–1.08)
Rural living (outside Gothenburg)	1.004 (0.79–1.28)	0.98 (0.79–1.22)
Occupational exposure to gas, dust or fumes	**1.76 (1.31–2.36)**	**1.67 (1.25–2.22)**
Asthma	**5.15 (3.67–7.23)**	**3.14 (2.35–4.19)**
Level of education:		
Primary / Secondary school	1.0	1.0
High school	1.35 (0.97–1.88)	**1.47 (1.10–1.97)**
University	**1.63 (1.09–2.44)**	**1.64 (1.12–2.41)**

Bold indicates statistical significance. aOR = adjusted odds ratio.

### Smoking

Current smoking decreased from 20.3% to 15.2% (p<0.001, aOR 0.70, 95% CI 0.60–0.82), significantly in females (23.5% to 16.2% p<0.001), but not males (p = 0.185). It was more common among females than males in 2008 (23.5% vs 16.2% p<0.001), with no significant difference in 2016 (16.2% vs 14.0%, p = 0.141). Ever smoking decreased from 25.2% to 20.1% (p<0.001, aOR 0.73, 95% CI 0.64–0.85), significantly in females (29.3% to 20.8%, p<0.001), but not males (19.8% to 19.2%, p = 0.776).

Respondents started smoking at a younger age in 2008 than 2016 (mean 15.3 vs 16.05 years, p<0.001), but the change was only significant in females (mean 14.9 vs 16.0 years, p<0.001), not males (mean 16.1 vs 16.1 years, p = 0.963). Females also started smoking earlier than males in 2008 (p<0.001), but not 2016 (p = 0.761). No overall difference in heavy smoking was seen in current smokers (47.5% vs 42%, p = 0.127). It reduced among females (51.9% vs 41.4%, p = 0.022), but not males (p = 0.545).

### Snus, e-cigarettes and nicotine

Ever use of snus decreased from 13.7% to 11.5% (p = 0.028), significantly in males (24.2% to 19.8%, p = 0.019). There was no significant change in the current use of snus, overall (9.8% to 8.6%, p = 0.151) or by males (18.5% to 15.6% p = 0.091). No significant difference was seen among females for ever use (5.8% to 5.3%, p = 0.606) or current use (3.3% to 3.4%, p = 1.0).

The use of e-cigarettes was only reported in 2016. The prevalence was 4.8%, mainly among males (6.6% vs 3.4%, p = 0.001). Most were also current smokers (60.7%), followed by non-smokers (28.6%) and ex-smokers (10.7%). E-cigarette users were more often heavy tobacco smokers compared to smokers who were non-users (67.2% vs 36.5%, p<0.001). Current snus use was more common among e-cigarette users than non-users (19.6% vs. 8.2%, p<0.001).

Ever nicotine use (33.3% to 27%, p<0.001) and current nicotine use (27.3% to 22.6%, p<0.001) was reduced, when stratified significantly among females (31.4% to 23.3%, p<0.001 and 25.4% to 18.9%, p<0.001, respectively). Males used significantly more nicotine than females in both 2008 (30.0% vs. 25.4% p<0.05) and 2016 (27.1 vs 18.9%, p<0.05),

## Discussion

This repeated, cross-sectional questionnaire-based study found that current asthma increased among young adults in Western Sweden from 2008–2016, particularly among male smokers and males without allergic rhinitis (AR). Growing up on a farm reduced the risk of asthma in both years. AR was stable, while tobacco smoking and nicotine use decreased, especially among females. Most e-cigarette users were also tobacco smokers.

This increase in asthma contradicts the plateau reported by several western countries [3, 4], but supports other findings [15, 16], which suggest that the asthma epidemic has not yet ended. However, AR had levelled off and our study did not confirm previous reports about increasing AR among children and adults [4, 10, 16]. The absence of a simultaneous increase in asthma and AR, combined with an increase in asthma without AR, indicates that AR could not explain the increase in asthma in our study. However, asthma and allergic diseases are closely related [9] and have been associated with a family history of either of them [3], indicating a genetic predisposition, as confirmed by our study. The close link between AR and asthma is clear, but it did not seem to drive the increasing prevalence of asthma in this young population.

In our study we could confirm a protective effect of living on a farm on both asthma and ever AR. Previous reports on the 2008 cohort showed a protective effect for asthma in 16–20 years old [3] and a reduced risk of AR throughout life, with the strongest effect seen in those aged 16–30 [13]. It could be argued that families with a history of asthma or allergy are unlikely to be farmers, but in our study this effect was independent of heredity in both cohorts, indicating a true protective correlation.

Tobacco smoking has also been identified as a risk factor for asthma and adverse respiratory symptoms [7, 17], but we found that these symptoms seemed to increase, despite reduced smoking rates, indicating a true increase in asthma. Smoking decreased significantly, and was not a risk factor for current asthma, but the increase in asthma was most prominent in male smokers. Thus, the overall increase in asthma in our study could not be attributed to either increasing AR or smoking. However, numerous lifestyle and dietary factors, have been associated with asthma and could have contributed to the increase [18, 19]. One study suggested that increased awareness of asthma and prescribing policies could partially explain increases in asthma and allergic diseases [20]. The simultaneous increase in relevant adverse respiratory symptoms in our study makes this less likely.

Given the worryingly high number of young female smokers in 2008 [3, 21], the reduction in 2016, especially among females, was very positive and in line with other Swedish reports [4, 7]. Females started smoking later in 2016 than 2008, with fewer heavy smokers. Moreover, overall nicotine use declined significantly, suggesting that tobacco smoking was not replaced. E-cigarettes have only been available in the last decade and only 1–4% of people use them on a regular basis [22, 23]. Our results suggest a higher prevalence of e-cigarette use among young adults (4.8%) than in the general population, especially among males.

There is ongoing controversy about whether e-cigarettes are a promising solution to the tobacco epidemic or a new potential danger [24]. Most of the e-cigarette users in our study were current, and mainly heavy, tobacco smokers as reported by other studies [23, 25]. E-cigarettes may replace conventional smoking in situations where it is not allowed or acceptable. Our study suggests they lead to nicotine dependence, rather than quitting.

### Strengths and limitations

The main strengths of our study were the two large cross-sectional cohorts, who were randomly selected and separated in time by eight years, as well as they shared the same age range and demographics. This added to the reliability of our analyses, as there was sufficient time to observe important changes.

The limitations included the possible validity of answers, which is common with epidemiological studies based on postal questionnaires. However, we used well-validated questionnaires as other studies did, which made comparisons possible [14, 26].

The response rate was lower in 2016, compared to 2008, a decrease over time described also by others [27, 28]. However, the response rate in our and other Nordic studies are still higher than in studies from other parts of the world [29]. Even so, the response rate in our young population was lower than in the whole population [8, 14, 30] and the response rate may have biased the prevalence estimates. Non-responders have generally been described as to lower the prevalence estimates [31] with responders being more prone to have symptoms and higher morbidity. This would implicate an overestimation of increase in prevalence and underestimations of reductions over time as a result of decreasing response rate. However, a non-responder study on the 2008 cohort failed to show any significant differences in diseases or symptoms [30], in accordance with other Nordic non-responder studies [28, 32], and indications are the same in the 2016 cohort [8]. When comparing basic demographics in the two cohorts we found no significant differences regarding possible confounders as age, gender, farm living, employment, and educational level. A difference was noted regarding urban living and family history. In the multivariate analyses we adjusted for several of these factors, including urban living and family history. This supports a true difference in the prevalence of asthma and AR between 2008 and 2016 in our study, but a possible bias need to be considered before generalising the results.

In addition, the non-responder study of the 2008 cohort reported lower participation by younger males and smokers [30]. This may also have affected prevalence estimates among these groups. However, the estimations of smoking in our study is supported by findings in other Swedish reports, arguing for a true reduction over time [4, 7].

## Conclusion

Current asthma increased from 2008–2016, especially among male smokers and males without AR. In contrast, current AR levelled off and tobacco smoking decreased, especially among females. The increasing prevalence of asthma could not be explained by trends in AR or smoking habits. Farm living remained a protective factor for asthma. Finally, our study indicated that e-cigarettes supported smoking rather than quitting, as most users were also heavy tobacco smokers.

## Supporting information

S1 Table Questions for outcome variables, respiratory symptoms, demographics and covariates. (PDF)

S2 Table Univariate risk factors for current asthma in 2008 and 2016. (PDF)

S3 Table The prevalence of respiratory symptoms during the last 12 months, by gender, in 2008 and 2016. (PDF)
