# Supplementary material for: Asthma increased in young adults from 2008–2016 despite stable allergic rhinitis and reduced smoking
Source: PLoS One. 2021 Jun 24;16(6):e0253322. doi: 10.1371/journal.pone.0253322 (PMC8224942; doi:10.1371/journal.pone.0253322)
Supplement: S1 Table — (PDF) [file pone.0253322.s001.pdf]

**Symtom**	**Respective questions**
Questions regarding asthma<br>‘	Have you ever been diagnosed as having asthma by a doctor?<br>Have you now or have you ever had asthma?<br>Do you use asthma medication (on a regular basis or as needed)?<br>Have you had asthma symptoms within the last 12 months?<br>Do you usually have wheezing or whistling in your chest when breathing?<br>Have you had whistling or wheeze in the chest at any occasion in the last 12 months?
Current Allergic rhinitis	Have you had problems with AR during the last 12 months?
Ever Allergic rhinitis	Have you now or have you ever had allergic eye or nose problems (hay fever)
Attacks of shortness of breath	Do you presently have, or have you had in the last 10 years, asthma symptoms and have you had these symptoms within the last 12 months?
Long-standing cough	Have you had longstanding cough during the last year?
Sputum production	Do you usually have phlegm when coughing or do you have phlegm in the chest which is difficult to bring up?
Chronic productive cough	Sputum production for at least 3 months during two subsequent years?
Wheeze with breathlessness	Have you had whistling or wheezing in the chest at any occasion during the last 12 months and have you been at all breathless when you had wheezing in the chest?
Any wheeze	Have you had whistling or wheeze in the chest at any occasion in the last 12 months?
Recurrent wheeze	Do you usually have wheezing or whistling in your chest when breathing?
Wheeze without cold	Have you had whistling or wheezing in the chest at any occasion during the last 12 months and have you had this wheezing or whistling in your chest when you have not had a cold?
Waking with tight chest	Have you waked up with tightness in the chest at any occasion in the last 12 months?
Dyspnoea	Do you usually get breathless when you walk on level ground with people your own age?

Demographics and covariates	Respective questions
Family history of asthma or allergy	’Have any of your parents or siblings ever had asthma or allergic eye / nose problems (hay fever) or both asthma and hay fever?
Occupational exposure to gas, dust or fumes	Have you been exposed on a large scale to dust, gases or fumes at work?
Unemployment	Not being currently employed or a student
Educational level	Which is the highest level of education that you have?’’ Responders divided into: ‘’primary / secondary school’’, ‘’high school’’ and ‘’university’’
E-cigarette user	Do you use e-cigarettes?
Current snuff use	Do you currently use snuff?
Ever snuff use	Have you ever used snuff during at least 6 months?
Number of cigarettes/day	How many cigarettes do you smoke per day?’’ Responders divided into: ‘’less’’ or ‘’more than 5 cigarettes per day’’
Current smoking	Smoking during the last 12 months
Ex-smoking	Having stopped smoking at least 12 months prior to the survey
Growing up on a farm	Have your family had a farm during your first 5 years of life
