# Supplementary material for: Asthma increased in young adults from 2008–2016 despite stable allergic rhinitis and reduced smoking
Source: PLoS One. 2021 Jun 24;16(6):e0253322. doi: 10.1371/journal.pone.0253322 (PMC8224942; doi:10.1371/journal.pone.0253322)
Supplement: S2 Table — (PDF) [file pone.0253322.s002.pdf]

S2 Table. Univariate risk factors for current asthma in 2008 and 2016.

Variable	2008 Current asthma % (n) Total 9.3 (199)	2008 No current asthma % (n) Total 90.7 (1944)	2008 OR (95% CI)	2016 Current asthma % (n) Total 11.5 (286)	2016 No current asthma % (n) Total 88.5 (2198)	2016 OR (95% CI)
Age 21-25 years	53.3 (106/199)	55.1 (1071/1942)	0.93 (0.69-1.24)	59.8 (171/286)	56.6 (1243/2198)	1.14 (0.89-1.47)
Female gender	64.3 (128/199)	55.8 (1085/1944)	**1.43 (1.05-1.93)**	62.2 (178/286)	56.2 (1235/2198)	1.29 (0.997-1.7)
Family history of asthma or allergy	75.7 (143/189)	46.6 (872/1870)	**3.56 (2.52-5.02)**	79.1 (204/258)	49.4 (1028/2079)	**3.86 (2.83-5.28)**
Current smoking	20.3 (40/197)	20.4 (393/1931)	0.997 (0.69-1.4)	21.4 (61/285)	14.4 (313/2172)	**1.62 (1.19-2.20)**
Rural living (outside Gothenburg)	37.7 (75/199)	42.8 (832/1944)	0.81 (0.60-1.09)	54.7 (156/285)	50.8 (1115/2193)	1.17 (0.91-1.50)
Growing up on a farm	2.6 (5/195)	7.2 (137/1912)	**0.34 (0.14-0.84)**	4.6 (13/280)	7 (151/2158)	0.65 (0.36-1.16)
Ever rhinitis	65.3 (130/199)	24.1 (469/1944)	**5.93 (4.35-8.08)**	62.6 (179/286)	28 (615/2198)	**4.31 (3.33-5.57)**
Current allergic rhinitis	58.8 (117/199)	18.8 (365/1944)	**6.17 (4.55-8.37)**	51 (146/286)	20.9 (459/2198)	**3.95 (3.07-5.09)**
Level of education:						
Primary / Secondary school	18.9 (37/196)	19.8 (374/1892)	1.0	18.6 (52/280)	20.8 (453/2177)	1.0
High school	54.1 (106/196)	54.8 (1036/1892)	1.03 (0.70-1.53)	63.2 (177/280)	55.7 (1213/2177)	1.27 (0.92-1.76)
University	27 (53/196)	25.5 (482/1892)	1.11 (0.72-1.73)	18.2 (51/280)	23.5 (511/2177)	0.87 (0.58-1.31)
Unemployed	4.6 (9/197)	4.7 (91/1919)	0.96 (0.48-1.94)	3.9 (11/283)	4.3 (94/2186)	0.90 (0.48-1.70)
Occupational exposure to gas, dust or fumes	18.6 (37/199)	17 (330/1944)	1.12 (0.77-1.63)	18.9 (54/286)	12.8 (282/2198)	**1.59 (1.15-2.19)**

Bold indicates statistical significance. OR=odds ratio.
