# Supplementary material for: Asthma increased in young adults from 2008–2016 despite stable allergic rhinitis and reduced smoking
Source: PLoS One. 2021 Jun 24;16(6):e0253322. doi: 10.1371/journal.pone.0253322 (PMC8224942; doi:10.1371/journal.pone.0253322)
Supplement: S3 Table — (PDF) [file pone.0253322.s003.pdf]

S3 Table. The prevalence of respiratory symptoms during the last 12 months, by gender, in 2008 and 2016.

	Females			Males		
	**2008**	**2016**		**2008**	**2016**	
**Respiratory symptoms**	**% (n)**	**% (n)**	**p-value**	**% (n)**	**% (n)**	**p-value**
Attacks of shortness of breath	11.8 (143/1213)	14 (198/1410)	0.091	6.6 (61/930)	9.7 (104/1071)	**0.011**
Any wheeze	19.1 (232/1213)	20.9 (296/1413)	0.261	11.5 (107/930)	16.5 (177/1071)	**0.001**
Wheezing with breathlessness	12.7 (154/1213)	13.3 (187/1410)	0.684	7 (65/930)	10 (107/1071)	**0.020**
Wheezing without cold	10.5 (127/1213)	10.7 (151/1410)	0.849	5.7 (53/930)	9 (96/1071)	**0.006**
Waking with tight chest	12.7 (154/1213)	12.7 (180/1413)	1.000	5.6 (52/930)	10.8 (116/1071)	**<0.001**
Long-standing cough	16 (194/1213)	14.6 (206/1412)	0.327	11.5 (107/930)	15.9 (170/1071)	**0.005**

Bold indicates statistical significance.
